# Supplementary material for: Epidemiology of leptospirosis in Tanzania: A review of the current status, serogroup diversity and reservoirs
Source: PLoS Negl Trop Dis. 2021 Nov 16;15(11):e0009918. doi: 10.1371/journal.pntd.0009918 (PMC8631673; doi:10.1371/journal.pntd.0009918)
Supplement: S1 Table — (DOCX) [file pntd.0009918.s001.docx]

S1­ Table. Summary of the papers included in this review of leptospirosis in Tanzania 1997-2019 including year of research, study design, geographical location, target populations, diagnostics tests, and results for each study

| **Ref** | **Year of study** | **Type of study** | **Human** | **Animal** | **Region** | **Culture** | **Serology** | **PCR** | **Serogroup (serovar) screened by MAT in people** | **Serogroup (serovar) screened by MAT in animals** |
| --- | --- | --- | --- | --- | --- | --- | --- | --- | --- | --- |
| [1] | 2013-2014 | Cross-sectional (village and abattoir) |  | Cattle n=452  Goats n=167  Sheep  n=89  Rodents n=384 | Kilimanjaro | Cattle 4/100  Goats  0/49  Rodents  0/98 |  | *lip*L32  Cattle 32/452 (7.08%)  Goats  2/167 (1.2%)  Sheep  1/89  (1.12%)  Rodents  0/384 (0%) |  | Not examined |
| [2] | 2007-2008**  2012-2014* | Hospital (febrile patients) | People  n=1849 |  | Kilimanjaro | People  0/1294 | MAT 81/1225 | *lip*L32  4/109 | Described by [4] & [20] |  |
| [3] | 2012-2013*** | Cross-sectional | People  n=267 | Cattle  n=1103  Goats  n=248  Buffalo  n=38  Zebra  n=2  Lions  n=2  Rodents  n=207  Shrews  n=11 | Katavi |  | MAT  People  80/267  29.96%  Cattle  335/1103  30.37%  Goats  21/248  8.47%  Buffalo  11/38  28.95%  Rodents  42/207  20.29%  Shrews  1/11  9.09%  Lion  1/2  Zebra  0/2 |  | - Sejroe (Hardjo) - Hebdomadis (Hebdomadis) - Grippotyphosa (Grippotyphosa) - Icterohaemorrhagiae (Sokoine) - Australis (Lora) - Ballum (Kenya) - Canicola (Canicola) ND | - Sejroe (Hardjo) - Hebdomadis (Hebdomadis) - Grippotyphosa (Grippotyphosa) - Icterohaemorrhagiae (Sokoine) - Australis (Lora) - Ballum (Kenya) ND - Canicola (Canicola) ND |
| [4] | 2007-2008** | Hospital (Febrile children and adults) | Children and adults  n=831 |  | Kilimanjaro |  | MAT  346/831 (41.6%)  (seropositive) |  | - **Australis (Australis, Bratislava)** - **Autumnalis (Autumnalis)** - Ballum (Ballum) - Bataviae (Bataviae) - **Canicola (Canicola)** - **Celledoni (Celledoni)** - Cynopteri (Cynopteri) - **Djasiman (Djasiman)** - **Grippotyphosa (Grippotyphosa)** - **Hebdomadis (Borincana)** - **Icterohaemorrhagiae (Mankarso, Icterohaemorrhagiae)** - Javanica (Javanica) - **Mini (Georgia)** - Pomona (Pomona) - **Pyrogenes (Pyrogenes, Alexi)** - Sejroe (Wolfii) - **Tarassovi (Tarassovi)**   All serogroups were detected. The predominant serogroups detected are in bold |  |
| [5] | 2007-2008** | Hospital (Febrile children and adults) | Children and adults  n=831 |  | Kilimanjaro |  | MAT  70/831 (confirmed and probable cases) |  | Described by [4] |  |
| [6] | 2007-2008** | Hospital (children and adults) | n=870 |  | Kilimanjaro |  | MAT  42/588 |  | Described by [4] |  |
| [7] | 2013-2014 | Hospital (febrile adults) | Adults  n=519 |  | Dar es Salaam |  |  | PCR  1/519 |  |  |
| [8] | 2013 | Hospital (febrile children) | Children  n=370 |  | Morogoro |  | Acute  ELISA  43/370 (11.6%)  MAT  26/200 (13%) |  | - Icterohaemorrhagiae (Sokoine) - Ballum (Kenya) - Grippotyphosa (Grippotyphosa) - Hebdomadis (Hebdomadis) - Australis (Lora) - Sejroe (Hardjo) ND |  |
| [9] | 2007-2008** | Hospital (febrile children and adults) | Children and adults  n=453 |  | Kilimanjaro |  | MAT  40/453 (8.8%)  (confirmed cases) |  | Described by [4] |  |
| [10] | 2008 | Hospital (children) | Children  n=1005 |  | Morogoro and Dar es Salaam |  | MAT  4/1005 |  | - Icterohaemorrhagiae (Icterohaemorrhagiae) - Sejroe (Hardjo) |  |
| [11] | 2014-2015 | Hospital based (febrile children and adults) | Children and adults  n=842 |  | Morogoro |  |  | TAC  22/842 |  |  |
| [12] | 2014 | Hospital based (febrile children and adults) | Children and adults n=191 |  | Morogoro |  |  | TAC  3/191 |  |  |
| [13] | 2005 | Case control |  | Cattle  n=80 | Tanga |  | MAT  Cattle  17/80 (21.3%) |  |  | - Sejroe (Hardjo), - Icterohaemorrhagiae (Icterohaemorrhagiae) - Grippotyphosa (Grippotyphosa) - Pomona (Pomona) - Canicola (Canicola) ND |
| [14] | 2007-2008 | Cross-sectional |  | Rodents &  Insectivores  n=350 | Morogoro |  | MAT  62/348 (17.8%) |  |  | - Icterohaemorrhagiae (Sokoine) - Pomona (Pomona) - Sejroe (Hardjo) - Ballum (Ballum) - Grippotyphosa (RM4) - Canicola (Canicola) |
| [15] | No date | Cross-sectional |  | Rodents  n=70 | Morogoro |  | MAT  16/70 (22.9%) |  |  | - Icterohaemorrhagiae (Sokoine) - Ballum (Kenya) - Australis (Lora) |
| [16] | 2007-2008 | Cross-sectional |  | Pigs  n=385 | Morogoro | Pigs  2/236 | MAT  17/385 (4.4%) |  |  | - Pomona (Pomona) - Ballum (Ballum) - Icterohaemorrhagiae (RM1) - Grippotyphosa (RM4) ND - Tarassovi (Tarassovi) ND - Sejroe (Hardjo) ND |
| [17] | 1996 | Cross-sectional | People  n=375 | Cattle  n=374 blood  n=1021 urine  Dogs  n=208  Rodents  n=537 | Morogoro, Kilimanjaro, Sigida, Mwanza, Mbeya, Tanga, Dar es Salaam | Cattle  7/1021 | MAT  People  1/375  Cattle  28/374  Dogs  80/208  Rodents  10/537 |  | - Icterohaemorrhagiae (Icterohaemorrhagiae) ND - Grippotyphosa (Grippotyphosa) | Rodents   - Icterohaemorrhagiae (Icterohaemorrhagiae)   Cattle   - Sejroe (Hardjo) - Pyrogenes (Pyrogenes)   Dogs   - Canicola (Canicola) - Icterohaemorrhagiae (Icterohaemorrhagiae) |
| [18] | No date | Targeted |  | Giant pouched rats | Morogoro |  | CAAT |  |  |  |
| [19] | 2007-2008**  2012-2014* | Hospital (febrile patients) | Children and adults  n=1115 |  | Kilimanjaro |  | MAT  19/1115 (1.7%) |  | Described by [4] & [20] |  |
| [20] | 2012-2014* | Hospital (febrile children and adults) | Children and adults  n=1293 |  | Kilimanjaro |  | MAT  Single sample  252/1293 (19.5%)  Acute leptospirosis  24/1293 (1.8%) |  | - Australis (Australis, Bratislava) - Autumnalis (Autumnalis) - Ballum (Ballum) ND - Bataviae (Bataviae) - Canicola (Canicola) - Celledoni (Celledoni) - Cynopteri (Cynopteri) ND - Djasiman (Djasiman) - Grippotyphosa (Grippotyphosa) - Hebdomadis (Borincana) - Icterohaemorrhagiae (Mankarso, Icterohaemorrhagiae) - Javanica (Javanica) ND - Mini (Georgia) - Pomona (Pomona) ND - Pyrogenes (Pyrogenes, Alexi) - Sejroe (Wolffi) - Tarassovi (Tarassovi) |  |
| [21] | 2016-2017 | Hospital (febrile patients) | Children and adults n=104 |  | Arusha |  | MAT  6/104 |  | - Australis (Australis, Bratislava) - Autumnalis (Autumnalis) ND - Ballum (Ballum) ND - Bataviae (Bataviae) ND - Canicola (Canicola) ND - Celledoni (Celledoni) ND - Cynopteri (Cynopteri) ND - Djasiman (Djasiman) - Grippotyphosa (Grippotyphosa) ND - Hebdomadis - (Borincana) ND - Icterohaemorrhagiae (Mankarso, Icterohaemorrhagiae) - Javanica (Javanica) - Mini (Georgia) ND - Pomona (Pomona) ND - Pyrogenes (Pyrogenes, Alexi) ND - Sejroe (Wolfii) ND - Tarassovi (Tarassovi) |  |
| [22] | No date | Not stated |  | Rodents n=20  Insectivores  n=7 | Morogoro | Rodents  0/20  Insectivores  2/7 | MAT  Rodents  0/20  Insectivores  0/7 | PCR  Rodents 1/20  Insectivores 2/7 |  | - Icterohaemorrhagiae (Sokoine) ND - Ballum (Kenya) ND - Hebdomadis (Hebdomadis) ND - Pomona (Pomona) ND - Sejroe (Hardjo) ND |
| [23] | No date | Targetted |  | Cattle | Morogoro |  | CAAT |  |  |  |
| [24] | 2013 | Cross sectional |  | Bats  n=36 | Morogoro |  | MAT  7/36 (19.4%) |  |  | - Icterohaemorrhagiae (Sokoine) - Ballum (Kenya) - Hebdomadis (Hebdomadis) ND - Australis (Lora) - Canicola (Canicola) ND - Pomona (Pomona) ND |
| [25] | 2003 | Cross-sectional |  | Fish  n=48 | Morogoro |  | MAT  26/48 (54.2%) |  |  | - Ballum (Kenya), - Icterohaemorrhagiae (Sokoine) - Pomona (Pomona) - Hebdomadis (Hebdomadis) ND |
| [26] | 2012-2013 | Cross-sectional |  | Rodents  n=89  Insectivores  n=1 | Morogoro |  | MAT  Rodents  23/89 (25.8%)  Insectivores  1/1 |  |  | - Icterohaemorrhagiae (Sokoine) - Ballum (Kenya) - Australis (Lora) - Canicola (Canicola) - Hebdomadis (Hebdomadis) - Pomona (Pomona) ND |
| [27] | 1996-2006 | Cross-sectional and hospital | People  Culture  n=589  (Abattoir workers n=83,  Patients n=506)  MAT  Patients  n=400 | Culture  Cattle  n=1021  Pigs  n=236  Giant pouch rats  n=285  Field rats  n=1382  Insectivore  n=298  MAT  Goats and sheep  n=100  Pigs  n=100  Dogs  n=100  Cats  n=64  Small rodents  n=500  Small rodents (different area)  n=90  African giant rats  n=65  Insectivores  n=4 | Morogoro | People  4/589  Cattle  7/1021^++^  Pigs  2/236++  Giant pouch rats  24/285  Field rats  8/1382  Insectivores  11/298 | Patients  70/400  Goats and sheep  38/100^+^  Pigs  41/100^+^  Dogs  39/100^+^  Cats  9/64^+^  Small rodents  25/500^+^  15/90^+^  African giant rats  10/65^+^  Insectivores  1/4^+^ |  | - Icterohaemorrhagiae (Sokoine, Mwogolo) - Ballum (Kenya) - Grippotyphosa (Grippotyphosa) - Canicola (Canicola) - Pomona (Pomona) - Sejroe (Hardjo) | - Icterohaemorrhagiae (Sokoine, Mwogolo) - Ballum (Kenya) - Australis (Lora) - Grippotyphosa (Grippotyphosa) - Hebdomadis (Hebdomadis) - Canicola (Canicola) - Pomona (Pomona) - Sejroe (Hardjo)   ^+^Results for Icterohaemorrhagiae  ++Cattle and pig results for results for isolation are also reported by [17] and [16] |
| [28] | No date | Cross-sectional (risk occupations) | People  n=455 | Rodents and insectivores  n=31 | Kagera | Rodent and Insectivores  0/31 | MAT  People 72/455 (15.8%)  Rodents and insectorvores  4/24 |  | - Icterohaemorrhagiae (Sokoine) - Australis (Lora) - Grippotyphosa (Grippotyphosa) ND - Pomona (Pomona) - Hebdomadis (Hebdomadis) - Ballum (Kenya) | - Icterohaemorrhagiae (Sokoine) - Australis (Lora) ND - Grippotyphosa (Grippotyphosa) - Pomona (Pomona) ND - Hebdomadis (Hebdomadis) ND - Ballum (Kenya) ND |
| [29] | 2017 | Cross-sectional (risk occupations) | People  n=250 |  | Mwanza |  | MAT  26/250 (10%) |  | - Icterohaemorrhagiae (Sokoine) - Grippotyphosa (Grippotyphosa) - Australis (Lora) - Ballum (Kenya) ND - Hebdomadis (Hebdomadis) ND |  |
| [30] | 2013-2014*** | Cross-sectional (community) | People n=267 |  | Katavi |  | MAT  80/267  29.9% | 16s  33/210  15.7% | - Sejroe (Hardjo) - Icterohaemorrhagiae (Sokoine), - Grippotyphosa (Grippotyphosa) - Hebdomadis (Hebdomadis) - Australis (Lora) - Ballum (Kenya)   Also described by [3] |  |
| [31] | 2016-2017 | Cross-sectional |  | Dogs  n=232 | Morogoro |  | MAT  22/232  9.5% |  |  | - Pomona (Pomona) - Icterohaemorrhagiae (Sokoine) - Grippotyphosa (Grippotyphosa) - Australis (Lora) - Ballum (Kenya) - Hebdomadis (Hebdomadis) ND |
| [32] | 2005 | Cross-sectional | People  n=199 |  | Tanga |  | MAT  30/199  15.1% |  | - Icterohaemorrhagiae (Icterohaemorrhagiae) - Sejroe (Hardjo) - Bataviae (Bataviae) - Tarassovi (Tarassovi) - Pomona (Pomona) - Ballum (Ballum) |  |
| [33] | 2003-2004 | Cross-sectional |  | Cattle  n=655 | Tanga |  | MAT  198/655  30.3% |  |  | - Sejroe (Hardjo) - Bataviae (Bataviae), - Tarassovi (Tarassovi) - Pomona (Pomona) |
| [34] | 2002-2004 | Cross-sectional (abattoir) |  | Cattle n=51 | Tanga |  | MAT &  Eiken latex agglutination  26/51 (51%) |  |  | - Sejroe (Hardjo) - Ballum (Ballum) ND - Icterohaemorrhagiae (Icterohaemorrhagiae) ND - Bataviae (Bataviae) - Tarassovi (Tarassovi) - Pomona (Pomona) ND |

*Studies reporting on the same sample [2,4–6,19]

**Studies reporting on the same sample [2,19,20]

*** Studies reporting on the same sample [3,30]

Ref - Reference

MAT – Microscopic agglutination test

CAAT – Cross agglutination absorption test

TAC – TaqMan Array Cards

ND – Not detected

References

1. Allan KJ, Halliday JEBB, Moseley M, Carter RW, Ahmed A, Goris MGAA, et al. Assessment of animal hosts of pathogenic Leptospira in northern Tanzania. Foley J, editor. PLoS Neglected Tropical Diseases. 2018 Jun;12(6):1–19.
2. Allan KJ, Maze MJ, Galloway RL, Rubach MP, Biggs HM, Halliday JEB, et al. Molecular detection and typing of pathogenic leptospira in febrile patients and phylogenetic comparison with leptospira detected among animals in Tanzania. American Journal of Tropical Medicine and Hygiene. 2020;103(4):1427–34.
3. Assenga JA, Matemba LE, Muller SK, Mhamphi GG, Kazwala RR, Mhamphi GG, et al. Predominant Leptospiral Serogroups Circulating among Humans, Livestock and Wildlife in Katavi-Rukwa Ecosystem, Tanzania. PLOS Neglected Tropical Diseases. 2015 Mar;9(3):e0003607.
4. Biggs HM, Bui DM, Galloway RL, Stoddard RA, Shadomy S V., Morrissey AB, et al. Leptospirosis among hospitalized febrile patients in northern Tanzania. American Journal of Tropical Medicine and Hygiene. 2011;85(2):275–81.
5. Biggs HM, Galloway RL, Bui DM, Morrissey AB, Maro VP, Crump JA. Leptospirosis and human immunodeficiency virus co-infection among febrile inpatients in northern Tanzania. Vector-Borne and Zoonotic Diseases. 2013 Aug;13(8):572–80.
6. Biggs HM, Hertz JT, Munishi OM, Galloway RL, Marks F, Saganda W, et al. Estimating Leptospirosis Incidence Using Hospital-Based Surveillance and a Population-Based Health Care Utilization Survey in Tanzania. PLoS Neglected Tropical Diseases. 2013;7(12):1–8.
7. Boillat-Blanco N, Mbarack Z, Samaka J, Mlaganile T, Kazimoto T, Mamin A, et al. Causes of fever in Tanzanian adults attending outpatient clinics: a prospective cohort study. Clinical Microbiology and Infection. 2021;27(6):913.e1-913.e7.
8. Chipwaza B, Mhamphi GG, Ngatunga SD, Selemani M, Amuri M, Mugasa JP, et al. Prevalence of Bacterial Febrile Illnesses in Children in Kilosa District, Tanzania. PLoS Neglected Tropical Diseases. 2015;9(5).
9. Crump JA, Morrissey AB, Nicholson WL, Massung RF, Stoddard RA, Galloway RL, et al. Etiology of Severe Non-malaria Febrile Illness in Northern Tanzania: A Prospective Cohort Study. PLoS Neglected Tropical Diseases. 2013;7(7):e2324.
10. D’Acremont V, Kilowoko M, Kyungu E, Philipina S, Sangu W, Kahama-Maro J, et al. Beyond Malaria — Causes of Fever in Outpatient Tanzanian Children. Vol. 370, New England Journal of Medicine. 2014. p. 809–17.
11. Hercik C, Cosmas L, Mogeni OD, Wamola N, Kohi W, Omballa V, et al. A diagnostic and epidemiologic investigation of acute febrile illness (AFI) in Kilombero, Tanzania. Schildgen O, editor. PLoS ONE. 2017 Dec;12(12):e0189712.
12. Hercik C, Cosmas L, Mogeni OD, Wamola N, Kohi W, Houpt E, et al. A combined syndromic approach to examine viral, bacterial, and parasitic agents among febrile patients: A pilot study in Kilombero, Tanzania. American Journal of Tropical Medicine and Hygiene. 2018 Feb;98(2):625–32.
13. Karimuribo ED, Swai ES, Kyakaisho PK. Investigation of a syndrome characterised by passage of red urine in smallholder dairy cattle in East Usambara Mountains, Tanzania. Journal of the South African Veterinary Association. 2008 Jun;79(2):89–94.
14. Katakweba AAS, Mulungu LS, Eiseb SJ, Mahlaba TATA, Makundi RH, Massawe AW, et al. Prevalence of haemoparasites, leptospires and coccobacilli with potential for human infection in the blood of rodents and shrews from selected localities in Tanzania, Namibia and Swaziland. African Zoology. 2012 Apr;47(1):119–27.
15. Katakweba A. Small Mammals in Fenced Houses as Source of Leptospirosis to Livestock Pets animals and Humans in Morogoro Municipality, Tanzania. Tanzania Veterinary Association Proceedings [Internet]. 2018;36(2018). Available from: https://www.ajol.info/index.php/tvj/article/view/194951%0Ahttps://tvj1.sua.ac.tz/index.php/TVJ/article/view/83
16. Kessy MJ, Machang’u RS, Swai ES. A microbiological and serological study of leptospirosis among pigs in the Morogoro municipality, Tanzania. Tropical Animal Health and Production. 2010 Mar;42(3):523–30.
17. Machang’u RS, Mgode G, Mpanduji D. Leptospirosis in animals and humans in selected areas of Tanzania. Belgian Journal of Zoology. 1997;127 Suppl(January):97–104.
18. Machang’u RS, Mgode GF, Assenga J, Mhamphi G, Weetjens B, Cox C, et al. Serological and molecular characterization of leptospira serovar Kenya from captive African giant pouched rats (Cricetomys gambianus) from Morogoro Tanzania. FEMS Immunology and Medical Microbiology. 2004;41(2):117–21.
19. Maze MJ, Biggs HM, Rubach MP, Galloway RL, Cash-Goldwasser S, Allan KJ, et al. Comparison of the Estimated Incidence of Acute Leptospirosis in the Kilimanjaro Region of Tanzania between 2007–08 and 2012–14. PLoS Neglected Tropical Diseases. 2016 Dec;10(12):1–15.
20. Maze MJ, Cash-Goldwasser S, Rubach MP, Biggs HM, Galloway RL, Sharples KJ, et al. Risk factors for human acute leptospirosis in northern Tanzania. Foley J, editor. PLoS Neglected Tropical Diseases. 2018 Jun;12(6):1–22.
21. Maze MJ. The impact of leptospirosis in Northern Tanzania [Internet]. University of Otago; 2019. Available from: https://ourarchive.otago.ac.nz/handle/10523/8838
22. Mgode GF, Mhamphi G, Katakweba A, Paemelaere E, Willekens N, Leirs H, et al. Pcr detection of Leptospira DNA in rodents and insectivores from Tanzania. Belgian Journal of Zoology. 2005;135(SUPPL.1):17–9.
23. Mgode GF, Machang’u RS, Goris MG, Engelbert M, Sondij S, Hartskeerl RA. New Leptospira serovar Sokoine of serogroup Icterohaemorrhagiae from cattle in Tanzania. International Journal of Systematic and Evolutionary Microbiology. 2006;56(3):593–7.
24. Mgode GF, Mbugi HA, Mhamphi GG, Ndanga D, Nkwama EL. Seroprevalence of leptospira infection in bats roosting in human settlements in Morogoro municipality in Tanzania. Tanzania Journal of Health Research. 2014;16(1):1–7.
25. Mgode GF, Mhamphi GG, Katakweba AS, Thomas M. Leptospira infections in freshwater fish in Morogoro Tanzania: A hidden public health threat. Tanzania Journal of Health Research. 2014;16(2):1–7.
26. Mgode GF, Katakweba AS, Mhamphi GG, Fwalo F, Bahari M, Mashaka M, et al. Prevalence of leptospirosis and toxoplasmosis: A study of rodents and shrews in cultivated and fallow land, Morogoro rural district, Tanzania. Tanzania Journal of Health Research. 2014 Jul;16(3):1–7.
27. Mgode GF, Machang’u RS, Mhamphi GG, Katakweba A, Mulungu LS, Durnez L, et al. Leptospira Serovars for Diagnosis of Leptospirosis in Humans and Animals in Africa: Common Leptospira Isolates and Reservoir Hosts. PLoS Neglected Tropical Diseases. 2015;9(12).
28. Mgode GF, Japhary MM, Mhamphi GG, Kiwelu I, Athaide I, Machang’u RS. Leptospirosis in sugarcane plantation and fishing communities in Kagera northwestern Tanzania. PLoS Neglected Tropical Diseases. 2019 May;13(5):1–12.
29. Mirambo MM, Mgode GF, Malima ZO, John M, Mngumi EB, Mhamphi GG, et al. Seroposotivity of Brucella spp. and Leptospira spp. antibodies among abattoir workers and meat vendors in the city of Mwanza, Tanzania: A call for one health approach control strategies. Foley J, editor. PLoS Neglected Tropical Diseases. 2018 Jun;12(6):39–52.
30. Muller SK, Assenga JA, Matemba LE, Misinzo G, Kazwala RR. Human leptospirosis in Tanzania: sequencing and phylogenetic analysis confirm that pathogenic Leptospira species circulate among agro-pastoralists living in Katavi-Rukwa ecosystem. BMC Infectious Diseases. 2016 Dec;16(1):273.
31. Said K, Bakari G, Machang’u R, Katakweba A, Muhairwa A. Seroprevalence of canine leptospirosis, in Urban and Periurban, Morogoro, Tanzania. African Journal of Microbiology Research. 2018;12(21):481–7.
32. Schoonman L, Swai ES. Risk factors associated with the seroprevalence of leptospirosis, amongst at-risk groups in and around Tanga city, Tanzania. Annals of Tropical Medicine and Parasitology. 2009 Dec;103(8):711–8.
33. Schoonman L, Swai ES. Herd- and animal-level risk factors for bovine leptospirosis in Tanga region of Tanzania. Tropical Animal Health and Production. 2010 Oct;42(7):1565–72.
34. Swai ES, Schoonman L. A survey of zoonotic diseases in trade cattle slaughtered at Tanga city abattoir: A cause of public health concern. Asian Pacific Journal of Tropical Biomedicine. 2012 Jan;2(1):55–60.
