# Supplementary material for: Epidemiology of leptospirosis in Tanzania: A review of the current status, serogroup diversity and reservoirs
Source: PLoS Negl Trop Dis. 2021 Nov 16;15(11):e0009918. doi: 10.1371/journal.pntd.0009918 (PMC8631673; doi:10.1371/journal.pntd.0009918)
Supplement: S2 Table — A) Prevalence of antibodies to Leptospira serogroups in people in cross-sectional studies; B) in febrile patients; C) in rodents; D) in cattle in Tanzania in leptospirosis papers published 1997–2021. (DOCX) [file pntd.0009918.s002.docx]

S2 Table. A) Prevalence of antibodies to *Leptospira* serogroups in people in cross-sectional studies; B) in febrile patients; C) in rodents; D) in cattle in Tanzania in leptospirosis papers published 1997-2021

| **Location, sample size and reference** | **Australis** | **Ballum** | **Djasiman** | **Grippotyphosa** | **Hebdomadis** | **Icterohaemorrhagiae** | **Sejroe** | **Tarassovi** |
| --- | --- | --- | --- | --- | --- | --- | --- | --- |
| **A) Cross-sectional** | | | | | | | | |
| Katavi n=267 [1] | 1.50 | 1.20 |  | 4.80 | 3.40 | 8.90 | 15.70 |  |
| Dar es Salaam n=375 [2] |  |  |  | 0.30 |  | 0.00 |  |  |
| Kagera n=455 [3] | 6.80 | 0.20 |  | 0.00 | 1.10 | 5.30 |  |  |
| Mwanza n=250 [4] | 2.00 | 0.00 |  | 1.20 | 0.00 | 7.20 |  |  |
| Tanga n=199 [5] |  | 0.50 |  |  |  | 5.50 | 3.00 | 1.00 |
| **Mean** | **3.43** | 0.48 | NT | 1.58 | 1.50 | **5.38** | **9.35** | 1.00 |
| **B) Hospital** | | | | | | | | |
| Kilimanjaro n=70 [6] | 30.00 | 0.00 | 2.80 | 1.40 | 1.40 | 6.00 | 0.00 | 1.40 |
| Morogoro n=26 [7] | 7.70 | 23.10 |  | 23.10 | 23.10 | 34.60 | 0.00 |  |
| Kilimanjaro n=24 [8] | 37.50 | 0.00 | 12.50 | 8.30 | 0.00 | 12.50 | 16.70 | 4.20 |
| Arusha n=6 [9] | 6.70 | 0.00 | 33.30 | 0.00 | 0.00 | 16.70 | 0.00 | 16.70 |
| **Mean** | **20.48** | 5.78 | **16.20** | 8.20 | 6.13 | **17.45** | 4.18 | 7.4 |
| **C) Rodents** | | | | | | | | |
| Katavi n=207 [10] | 18.80 | 0.00 |  | 0.50 | 0.00 | 1.90 | 0.00 |  |
| Morogoro n=70 [11] | 5.70 | 5.70 |  |  |  | 11.40 |  |  |
| Morogoro n=350 [12] |  | 0.90 |  | 1.40 |  | 10.30 | 1.10 |  |
| Morogoro, Tanga n=537 [2] |  |  |  |  |  | 1.80 |  |  |
| Morogoro n=20 [13] |  | 0.00 |  |  | 0.00 | 0.00 | 0.00 |  |
| Morogoro n=89 [14] | 9.00 | 2.20 |  |  | 1.10 | 16.90 |  |  |
| Kagera n=23 [3] | 0.00 | 0.00 |  | 4.30 | 0.00 | 8.70 |  |  |
| **Mean** | **8.38** | 1.47 | NT | 2.07 | 0.28 | **7.29** | 0.37 | NT |
| **D) Cattle** | | | | | | | | |
| Katavi n=1103 [10] | 0.80 | 0.00 |  | 4.80 | 7.70 | 4.70 | 17.60 |  |
| Tanga n=80 [15] |  |  |  |  | 2.50 | 3.80 | 12.50 |  |
| Kilimanjaro, Dar es Salaam, Mbeya=374 [2] |  |  |  |  |  |  | 5.60 |  |
| Mwanza, Mbeya=360 [2] |  |  |  |  |  |  |  |  |
| Tanga n=654 [16] |  |  |  |  |  |  | 15.00 | 12.20 |
| Tanga n=51 [17] |  |  |  |  |  |  | 29.00 | 18.00 |
| **Mean** | 0.80 | 0.00 | NT | 4.80 | 5.10 | 4.25 | **15.94** | **15.10** |

References

1. Muller SK, Assenga JA, Matemba LE, Misinzo G, Kazwala RR. Human leptospirosis in Tanzania: Sequencing and phylogenetic analysis confirm that pathogenic Leptospira species circulate among agro-pastoralists living in Katavi-Rukwa ecosystem. BMC Infect Dis [Internet]. 2016;16(1):1–9. Available from: http://dx.doi.org/10.1186/s12879-016-1588-x
2. Machang’u RS, Mgode G, Mpanduji D. Leptospirosis in animals and humans in selected areas of Tanzania. Belgian J Zool. 1997;127 Suppl(May 2016):97–104.
3. Mgode GF, Japhary MM, Mhamphi GG, Kiwelu I, Athaide I, Machang’u RS. Leptospirosis in sugarcane plantation and fishing communities in Kagera northwestern Tanzania. PLoS Negl Trop Dis. 2019;13(5):1–12.
4. Mirambo MM, Mgode GF, Malima ZO, John M, Mngumi EB, Mhamphi GG, et al. Seroposotivity of Brucella spp. and Leptospira spp. antibodies among abattoir workers and meat vendors in the city of Mwanza, Tanzania: A call for one health approach control strategies. PLoS Negl Trop Dis. 2018;12(6):39–52.
5. Schoonman L, Swai ES. Risk factors associated with the seroprevalence of leptospirosis, amongst at-risk groups in and around Tanga city, Tanzania. Ann Trop Med Parasitol. 2009;103(8):711–8.
6. Biggs HM, Bui DM, Galloway RL, Stoddard RA, Shadomy S V., Morrissey AB, et al. Leptospirosis among hospitalized febrile patients in northern Tanzania. Am J Trop Med Hyg. 2011;85(2):275–81.
7. Chipwaza B, Mhamphi GG, Ngatunga SD, Selemani M, Amuri M, Mugasa JP, et al. Prevalence of Bacterial Febrile Illnesses in Children in Kilosa District, Tanzania. PLoS Negl Trop Dis. 2015;9(5).
8. Maze MJ, Cash-Goldwasser S, Rubach MP, Biggs HM, Galloway RL, Sharples KJ, et al. Risk factors for human acute leptospirosis in northern Tanzania. PLoS Negl Trop Dis. 2018;12(6):1–22.
9. Maze MJ. The impact of leptospirosis in Northern Tanzania. University of Otago; 2019.
10. Assenga JA, Matemba LE, Muller SK, Mhamphi GG, Kazwala RR. Predominant Leptospiral Serogroups Circulating among Humans, Livestock and Wildlife in Katavi-Rukwa Ecosystem, Tanzania. PLoS Negl Trop Dis. 2015;9(3):1–14.
11. Katakweba A. Small Mammals in Fenced Houses as Source of Leptospirosis to Livestock Pets animals and Humans in Morogoro Municipality, Tanzania. Tanzania Vet Assoc Proc. 2018;36(2018).
12. Katakweba AAS, Mulungu LS, Eiseb SJ, Mahlaba TATA, Makundi RH, Massawe AW, et al. Prevalence of haemoparasites, leptospires and coccobacilli with potential for human infection in the blood of rodents and shrews from selected localities in Tanzania, Namibia and Swaziland. African Zool. 2012 Apr;47(1):119–27.
13. Mgode GF, Mhamphi G, Katakweba A, Paemelaere E, Willekens N, Leirs H, et al. Pcr detection of leptospira DNA in rodents and insecti-vores from Tanzania. Belgian J Zool. 2005;135(SUPPL.1):17–9.
14. Mgode GF, Katakweba AS, Mhamphi GG, Fwalo F, Bahari M, Mdangi M, et al. Prevalence of leptospirosis and toxoplasmosis: a study of rodents and shrews in cultivated and fallow land, Morogoro rural district, Tanzania. Tanzan J Health Res. 2014;16(3):1–7.
15. Karimuribo ED, Swai ES, Kyakaisho PK. Investigation of a syndrome characterised by passage of red urine in smallholder dairy cattle in East Usambara Mountains, Tanzania. J S Afr Vet Assoc. 2008;79(2):89–94.
16. Schoonman L, Swai ES. Herd- and animal-level risk factors for bovine leptospirosis in Tanga region of Tanzania. Trop Anim Health Prod. 2010;42(7):1565–72.
17. Swai ES, Schoonman L. A survey of zoonotic diseases in trade cattle slaughtered at Tanga city abattoir: A cause of public health concern. Asian Pac J Trop Biomed. 2012;2(1):55–60.
