# Supplementary material for: Epidemiology of leptospirosis in Tanzania: A review of the current status, serogroup diversity and reservoirs
Source: PLoS Negl Trop Dis. 2021 Nov 16;15(11):e0009918. doi: 10.1371/journal.pntd.0009918 (PMC8631673; doi:10.1371/journal.pntd.0009918)
Supplement: Attachment — Submitted filename: Response to Reviewers.docx [file pntd.0009918.s003.docx]

Shabani Kiyabo Motto

Tanzania Veterinary Laboratory Agency, Central Veterinary Laboratory,

131 Nelson Mandela Road

P.O Box 9254, Dar es salaam, Tanzania: Email: skymotto@gmail.com; skype: skymotto_1

July 12, 2021

The Editors

PLOS Neglected Tropical Diseases

[plosntds@plos.org](mailto:plosntds@plos.org)

Dear Editors:

Re: Resubmission of manuscript **Epidemiology of leptospirosis in Tanzania: A review of the current status, serogroup diversity and reservoirs** of reference number PNTD-D-21-00112R1.

Thank you for the chance to revise our review manuscript titled *Epidemiology of leptospirosis in Tanzania: A review of the current status, serogroup diversity and reservoirs.* We are grateful for the vigilant review and constructive suggestions. It is our believe that the manuscript is substantially improved after the edit suggested.

Following this latter, editor and reviewers comments with our responses in a track changes revision indicating where and how the text was refined. Changes made in the track changes manuscript. The revision has been developed in deliberation with all coauthors, and each author has given approval to the final changes of this revision. For this letter, all coauthors agreed and each remains valid.

Thank you for your consideration.

Sincerely,

**Shabani Kiyabo Motto**

Reviewer’s comments

| Reviewer #1: Thank you for the opportunity to review your systematic review. It was exciting to see a summary of recent research and a pleasure to read. I have some suggestions about areas in which the manuscript could be improved. | |
| --- | --- |
| MAJOR: | |
| Comments | Response |
| 1. Search strategy - You seem to be missing a number of relevant studies. It is not clear from the published search strategy why this is the case. When I use your search terms in Google I find additional references and understanding how/why these were excluded is important. For example: a. Allan KJ, Maze MJ, Galloway RL, Rubach MP, Biggs HM, Halliday JE, Cleaveland S, Saganda W, Lwezaula BF, Kazwala RR, Mmbaga BT. Molecular Detection and Typing of Pathogenic Leptospira in Febrile Patients and Phylogenetic Comparison with Leptospira Detected among Animals in Tanzania. The American Journal of Tropical Medicine and Hygiene. 2020 Oct;103(4):1427. - contains additional molecular data from Kilimanjaro Region b. Maze MJ. The Impact of Leptospirosis in Northern Tanzania: A Thesis Submitted for the Degree of Doctor of Philosophy at the University of Otago, Dunedin, New Zealand (Doctoral dissertation, University of Otago). This thesis contains a chapter on leptospirosis in the Ngorongoro Conservation Area. c. D'acremont V, Kilowoko M, Kyungu E, Philipina S, Sangu W, Kahama-Maro J, Lengeler C, Cherpillod P, Kaiser L, Genton B. Beyond malaria—causes of fever in outpatient Tanzanian children. New England Journal of Medicine. 2014 Feb 27;370(9):809-17. This study investigated leptospirosis among children with fever d. Boillat-Blanco N, Mbarack Z, Samaka J, Mlaganile T, Kazimoto T, Mamin A, Genton B, Kaiser L, D'Acremont V. Causes of fever in Tanzanian adults attending outpatient clinics: a prospective cohort study. Clinical Microbiology and Infection. 2020 Sep 4. This study used PCR to identify leptospirosis. | A literature search for this review was performed in late May 2020 and we added more literature manually through references. Thank you for your recommendations we have added these additional papers to the review. |
| 2. Leptospira exposure (ie seropositivity) vs leptospirosis (ie the acute disease) - It is important to clearly differentiate between the two as they are very different things. The lack of clarity is evident in line 299 - the Biggs 2011 study found 8.8% of patients hospitalized with a fever had acute leptospirosis with an additional 33.3% (ie 42.1% in total) seropositive. I suggest that you need to review all your papers regarding human infection again and consider how they should be interpreted. The interpretation is of course complex because acute leptospirosis would be unexpected in a cross sectional survey, and the incidence can only really be assessed by investigating those with a compatible illness. | Thank you for this suggestion. We have reviewed the papers and classified them as cross-sectional or hospital-based studies and then reported the results separately for seroprevalence and acute leptospirosis (Table 1 and table 2). We think this improves the report. The text has also been modified under the subheading **Leptospiral incidence and prevalence** |
| 3. Differentiating absent from not investigated. When considering MAT results I think you need to distinguish between serogroups that are not identified because they were not included in a panel and those that were included but tested negative. The entire section around predominant serovars needs reconsideration. In addition, Figure 5 is (I think) potentially misleading. As an alternative I think you should consider whether to report the prevalence of seropositivity by serogroup. | Thanks for this suggestion. We have addressed this issue regarding tested and undetected serogroups in Figure 3 and in the Supplementary Table 1. |
| MINOR | |
| General - Please review the written English within the paper - for example 'biasness' (line163) and numerous examples of disagreement between pronoun and noun (singular vs plural) and missing prepositions. Apologies for bringing this up, but the errors are to the point that they affect reading. | Thank you. We have reviewed the manuscript and revised the language accordingly. |
| INTRODUCTION: | |
| 1. There are some sentences (eg lines 82-83 about leptospirosis in Malaysia) and references (eg reference 5 the case report from Russia) that seemed of limited relevance to the topic. I suggest focusing comments and references towards Tanzania. | We have removed these comments and references. |
| 2. Lines 82-83 regarding modes of transmission fails to mention contact with a contaminated environment as a potential source of infection. | This has been added to the text Line 76 |
| Methods: | |
| 1. Including the search date is important (see above). | The literature search was conducted on 24^th^ May 2020. This has been added to the manuscript line 109. |
| 2. The ILS meeting is probably the most important scientific meeting for identifying leptospirosis abstracts and as far as I am aware it does not put the submitted abstracts on PubMed or Google. Did you have a strategy for reviewing these? | Thank you for this suggestion. We did check the ILS website and found abstracts available from 2019 meeting. There was an abstract presented by Allan et al. but it seems this work has subsequently been published and it was included in the review. |
| Results:  In addition to the 2 major comments above, |  |
| 1. Lines 246-247. The statement regarding the confirmed leptospirosis cases needs clarification. What is the definition? Where has the denominator come from? As above, a cross sectional seroprevalence study should not be expected to identify acute leptospirosis cases and shouldn't feature as part of a denominator. | We have reclassified the studies into cross sectional (seroprevalence) studies and hospital studies of febrile patients. For the cross sectional studies we have reported the number of seropositive individuals and the number tested. For the hospital studies we have reported the number of acute leptospirosis cases as defined by the authors and the number tested. This is also presented in Table 2 and the text under the heading **Leptospiral incidence and prevalence** in humans has been modified. |
| 2. Lines 256-263 - When considering the variation in estimated leptospirosis incidence you highlight study design, population sampling and study size, but neglect to consider variation in leptospirosis incidence. Is this not possible? | Line 316: We have added these details to the text. The annual incidence differences between the two studies reported may be due to differences in the population selected in the study, sample size or variation in leptospirosis incidence for unknown reasons. |
| Discussion: | |
| 1. Line 291 - While I am sympathetic to the perspective that leptospirosis doesn't get enough attention, I think the finding does need some support from your data. You have identified 30 studies - most published within the last decade. How much attention does it deserve, and from whom? | Line 298: This has been reworded. |
| 2. Lines 294-296 - The discussion about sources of leptospirosis does not mention rodents. Several authors have identified seropositive rodents and Mgode et al have isolated Leptospira from rodents. Totally discounting them seems a little premature. | Line 341: We have added a paragraph on rodents to the discussion and included the studies where Leptospira was detected by PCR and culture. |
| 3. Incidence of human leptospirosis - perhaps an alternative explanation of the data is that the incidence of leptospirosis varies by location and over time. The assertion that it has increased (line 298) is not supported by the only 2 actual datapoints on acute leptospirosis incidence (Biggs 2013 and Maze 2016). | We have removed this since there is no evidence that leptospirosis is increasing. |
| 4. Lines 324-327 - These seem to say that there has been only one study of rodents and it was negative. Your review shows this to be false. Mgode et al have identified Leptospira in rodents. Further you cite a number of technical errors that may have accounted for it. What about the possibility that Leptospira weren't (often) present in rats in Kilimanjaro region at the time of the study? It is notable that Allan et al did not trap many (if any) Mastomys species, whereas Mgode cultured Leptospira from them. There is precedent (a paper from Thailand last year I think) for low prevalence of leptospira carriage among peri-urban Rattus species, but high prevalence among rural rice field rats (Losea species from memory). | We have corrected this and thank you for the reference. Line 341-351 |
| 5. The paragraph on serologic approaches could more concisely state that MAT does not distinguish between infecting serovars. I think this is what you are getting at, but it is not clear. | We have reworded this to highlight the limitations of MAT line 321-323 |
| 6. The conclusion around RDTs is appealing, but the challenge is that (in my opinion at any rate) there isn't a good available RDT. While I appreciate that there isn't a lot of published data on this yet (there was an abstract at the ILS in 2017 reporting poor performance of lepto IgM RDTs in Kilimanjaro Region) your data doesn't really support this statement. | We have reworded this as a recommendation for RDT used in a clinical setting where MAT is not available as proposed in a recent policy brief Mgode 2017. |
| REFERNCES | |
| There are numerous errors (eg non italisized genus names) and a mish-mash of referencing styles and text (including all caps). | Indeed they were a mishmash. The references have been corrected |
| Reviewer #2: This is an interesting review on leptospirosis in Tanzania that could also be representing the situation in African region where this disease is highly neglected. The search seem to have skewed to mainly open access journals that full articles were retrievable and some important hosts have not been fully considered. Relevants comments can be found on respective sections and lines. Generally the work is worth and increases awareness and knowledge on this disease in the region. | |
| ABSTRACT | |
| Line 53: ...typhoid and brucellosis. should be typhoid, brucellosis and other diseases. Delete Despite in the next sentence in this line to start with "More than 250 pathogenic..... | This has been removed from the abstract. |
| Line 56: leptospira should be Leptospira | This has been corrected throughout. |
| INTRODUCTION | |
| Line 73: ....including Tanzania (10) where the disease.... This citation number 10 refers to Thailand not Tanzania. | This has been corrected. |
| Line 79: ......"incidences of 72-102 and 529 leptospiral cases....." a denominator for the 529 might be useful here. | This has been rewritten with the correct denominator. Line 90 |
| Line 82-83: .The high leptospiral cases in Malaysia.......296 deaths in 5 years: this sentence is not clear what it refers needs rephrasing and linking it well with the rest of contents in this paragraph. | The section has been deleted since it is irrelevant for Tanzania. |
| Line 87: .....non-pathogenic Leptospira (25, 26): Original references would fit better here, or add to these ones. | Thank you for noting this. The references have been amended. |
| METHODOLOGY -  RESULTS: | |
| Line 178: ....in humans (n=8) needs references. similarly, ...........two studies on animals (cattle, goat and rodents) - needs references as well.e.g. the two studies could be cited here. | This has been addressed. All the papers used are cited in the text or in the tables. |
| Line 226: ......serovars serotyped: should be serovars reported. | This has been corrected |
| Line 264: table 1: the locality named Moshi in column 3 should preferably be named as Moshi Kilimanjaro because the rest of the areas listed in this Table represent the regions while Moshi is an area in Kilimanjaro region. | Table 1 has changed but this has changed to the region name Kilimanjaro in Table 2 and 3 |
| Line 272: see comment on Moshi. should be Moshi Kilimanjaro. | This has been changed for consistency. |
| Line 277: .....Lemniscomys Griselda should be Lemniscomys griselda. the species name is small letter.  isolates from Tanzania (DOI: 10.1186/1476-0711-5-28). | This has been changed line 287 |
| Line 282: Table 2. omit DFM among the listed test - it is not a test but a tool for examining leptospires | This has been changed. |
| Row number 19 column no.5 the 455 are humans not rodents and shrews. In this study (39) there were rodents and shrews but the 455 are humans. | This has been corrected |
| For citation no. 59 (row no. 14 column 4 add MAT and culture. | These details have been removed from the Table and included in the Supplementary Table 1 |
| This Table can be enriched by including information from a study by Mgode et al (2015) published in PLOSNTD which gives insights on leptospirosis situation in broad range of reservoir hosts including data on cattle, cats, goats, sheep, pigs, rodents and shrews. manuscript and has a large/ in-depth report of Leptospira | Thank you for this suggestion we have included. Summary details are in Table 2 and 3 and Supplementary Table 1. |
| Another citation that reports genetic diversity of Leptospira isolates from Tanzania published by Ahmed et al (2006) could also add important information on Leptospira serovars reported from Tanzania which were described/identified using multilocus sequence typing.  This paper is missing in this manuscript and has a large/ in-depth report of Leptospira isolates from Tanzania (DOI: 10.1186/1476-0711-5-28). | We did review this paper but excluded it as not relevant to the review. The Tanzania isolates mentioned were used for developing an MLST and there was no information given regarding the context of their collection such as location or source. |
| DISCUSSION: | |
| Line 290.... it has not received sufficient attention.....This probably requires rephrasing because some work on leptospirosis in Tanzania has been used/cited in the National One Health strategic plan in Tanzania and East Africa community (specifically the work on leptospirosis in bats from Morogoro Tanzania, as well as publication on Leptospira serovar Sokoine from cattle. However, much would have been done given the amount of evidence that is available on leptospirosis and Leptospira from Tanzania. A policy brief on Leptospirosis in Tanzania is also in place (on researchgate) which highlights the situation of this disease in humans in Tanzania - probably contributes to the increasing awareness of non-malarial fevers. | The opening paragraph of the discussion has been rephrased. We have focused our conclusions on the unequal distribution of studies on leptospirosis in Tanzania and potential for control measures. |
| Line 297: For the past 9, ... this is incomplete, 9 what? years? | This has been corrected |
| Line 306: ...establish = established | This has been corrected |
| Line 368: ..... a list of candidate Leptospira serovars for diagnosis of leptospirosis using MAT in African region has been published (Mgode et al. 2015) which is based on data obtained from use of local serovars vs imported serovars. Similarly, policy brief (2016) recommending introduction of rapid diagnostic kits for leptospirosis has  been recommended for patients with non-malarial fevers (DOI: 10.13140/RG.2.2.13464.60165) could be relevant in this discussion line 373. | These details have been separated and elaborated upon in the discussion. Line 329- MAT panel and Line 368 regarding RDT |
| Line 460: citation no. 23: seem to be incomplete | This has been corrected |
| Reviewer #3: This manuscript synthesises what is known about the epidemiology of leptospirosis through a literature review. There is some confusion throughout the manuscript in the use of prevalence (presence of pathogen) and seroprevalence (presence of antibodies to the pathogen). The subject area is important and relevant and justifies publication. | |
| Line 14-15: "Leptospirosis is…" - Awkward sentence. Try rewording | This has been done |
| Line 34-36: Previously stated that 8 studies were undertaken in Kiliminjaro but this adds to 10 | Since the addition of more papers we have doubled checked all numbers. The details for each study are included in Supplementary Table 1. |
| Line 37: "Sejroe" not "Serjoe" | Corrected |
| Line 41-43: State the range in prevalence to provide some detail on how large the variation is | This has been added line 33 |
| Line 77: "subacute and long-lasting illness" - I'm not sure that these are the most appropriate references they just reference other studies which state this. Cite the initial studies where this was shown | Thank you. We have corrected this. |
| Line 81-83: Not sure why this is relevant | This has been deleted |
| Line 84: It is more than 250 serovars (as stated in abstract) | This has been corrected line 77 |
| Line 86: It is many more than 21 species. See recent paper <https://doi.org/10.1371/journal>. pntd.0007270 | This has been corrected line 79 |
| Line 169-178: When describing the number of studies focussing on each host type in each region these do not add up to the number of studies performed in each region (line 169-170) | This has been corrected. Since there is one study conducted in multiple regions the total will not equal the number of studies. We have addressed this in the text line 170 onwards. |
| Line 181: "serological diagnostic techniques" - many of these examples (culture, PCR) are not serological techniques | This has been corrected line 199 |
| Line 338-339: Risk of contamination of what? I'm not sure the author fully understands MAT | This has been removed. |
| Line 339-340: "MAT cannot be considered as a gold standard test" - but it is considered the gold standard serological test unless the author wishes to propose another test as gold standard | We have reworded this line 324 |
| Line 349-351: I think the discussion should include human exposure identified in clinical cases (fever) and human cases identified in surveillance (no fever). There are some serovars (Hardjo) that might not cause severe human disease. | This has been added. Both Table 1 and 2 have been separated in to prevalence studies and hospital based studies and the text under **Leptospiral incidence and prevalence.** |
| line 184 -186: This doesn't make sense. All the tests are diagnostic tests and I'm not sure what "widely recognised" means | This has been reworded |
| line 180-203: I don't think this figure is publication quality yet and I think it would be worth that providing references for each of the assays (perhaps as a supplementary table) | Thank you for the suggestion this has been removed and Supplementary Tables 1 and 2 added |
| line 209: If some serovars were frequently used then please list which ones | This has been clarified in the text line 215 – 221 and Figure 3. |
| line 218-219: provide a reference | Line 283: Referenced |
| line 219-221: This is for discussion not results Figure 4: I couldn't make sense of this figure. It needs to be improved | Line 218-221 moved to discussion  This figure has been removed. |
| Line 230-232: Confusing sentence. Not sure what the point is. Is it that lepto has been detected in every species except zebra or that it hasn't been detected in zebra because few animals were sampled? | This has been reworded line 242 |
| Line 246: I think this is misleading. It give the impression that all 7129 cases were suspected leptospirosis and only 576 were confirmed | We have distinguished between reports of seroprevalence and acute leptospirosis and modified these numbers (Table 1 and Table 2) and text under **Leptospiral incidence and prevalence** |
| Line 262-263: The most likely possibility is that lepto incidence truly did decrease | Line 314: The annual incidence differences between the two studies in reported incidence may be due to differences in the population selected, sample size or leptospirosis incidence decreased due to unknown reasons. |
| Line 279: presence of leptospires is not detected by MAT. Only leptospire antibodies are detected by MAT | This has been reworded |
| Line 297-298: "For the past 9.." something has been left out of this sentence | This has been reworded. |
| Line 306: "leptospirosis" not "Leptospirosis" | This has been corrected. |
| Line 306: "base level" - I am not clear that a base level prevalence is established in this study? To what does this refer? | This has been reworded line 309 |
| Line 313: To what does n=16 refer? | This has been corrected |
| Line 315: dogs aren't livestock | This has been corrected |
| Line 327: Again. The most likely cause of this finding is that the rodents weren't infected. It isn't uncommon as you point out. There doesn't have to be a methodology problem. | This has been corrected line 342-351 |
