# Supplementary material for: Epidemiology of leptospirosis in Tanzania: A review of the current status, serogroup diversity and reservoirs
Source: PLoS Negl Trop Dis. 2021 Nov 16;15(11):e0009918. doi: 10.1371/journal.pntd.0009918 (PMC8631673; doi:10.1371/journal.pntd.0009918)
Supplement: Attachment — Submitted filename: Response to reviewers.docx [file pntd.0009918.s004.docx]

Shabani Kiyabo Motto

Tanzania Veterinary Laboratory Agency, Central Veterinary Laboratory,

131 Nelson Mandela Road

P.O Box 9254, Dar es salaam, Tanzania: Email: skymotto@gmail.com; skype: skymotto_1

10-Sept-2021

The Editors

PLOS Neglected Tropical Diseases

[plosntds@plos.org](mailto:plosntds@plos.org)

Dear Editors:

Re: Resubmission of manuscript **Epidemiology of leptospirosis in Tanzania: A review of the current status, serogroup diversity and reservoirs** of reference number PNTD-D-21-00112R1.

On behalf of coauthors, we thank you all in the process of this review manuscript titled *Epidemiology of leptospirosis in Tanzania: A review of the current status, serogroup diversity and reservoirs.* We are grateful for all raised constractive comments. It is our believe that after this edit suggested the manuscript is substantially improved for publication.

Following this latter, editor and reviewers comments are imbedded below indicating where changes made in the track changes manuscript. All coauthors have given equal chance to approve final changes in this revision.

Thank you for your consideration.

Sincerely,

**Shabani Kiyabo Motto**

Reviewer’s comments

| Reviewer #1: Thank you for the opportunity to review this revised manuscript. My concerns from the initial paper have for the most part been addressed. Having read the revised manuscript, I have a small number of additional questions/ comments. | |
| --- | --- |
| Comments | Response |
| Introduction |  |
| Line 74: Including cattle, rodents and pigs - all true. Is there a role for dogs, sheep or goats? | Yes, we have added these details. Line 75 |
| Methods |  |
| Search strategy - I worry that the PubMed search strategy is too narrow. That it only identified 47 articles compared to 3720 identified in Google attests to that. It is perhaps not feasible to re-engineer your searches, but perhaps acknowledging the discrepancy between identified articles in Google vs PubMed and the potential to have missed articles that were not identified by the Google search would be appropriate. | Thanks for your comments. We consider that the PubMed search is an accurate representation of the articles published regarding leptospirosis in Tanzania. The over-representation of articles in the Google scholar search was because the search engine detected articles that mentioned Tanzania in the text of the paper. In this case any publication that had cited a paper from Tanzania was included in the search results. Many of the papers detected were reporting leptospirosis in another country. We have added these details to the text. Line 146 |
| Discussion |  |
| The claim of primacy isn't entirely true. What about Allan et al and De Vries et al who both conducted systematic reviews of the entire continent. Your review provides greater detail for Tanzania - but isn't the first to address the topic. | We have addressed this in the discussion 314 |
| Reviewer #3: Dear authors  Thank you for your revisions. Please see below a few suggestions. | |
| Line 81-83: Check this. There are 17 species in P1 and 21 in P2 | This has been corrected. Line 81 |
| Line 87: "small prevalence" should be "low prevalence" | We have reworded this. Line 88 |
| Lines 178-184: These sentences are more discussion than results | These have been moved to the discussion lines: 320 |
| Lines 208-209: Molecular typing is not a diagnostic technique. It is way of identifying genetic diversity | This has been reworded line 216 |
| Line 215: Should be Figure 3 | Changed line 227 |
| Figure 3: "Javonica" should be "Javanica" | This has been changed |
| Line 227: "serovars" should be "serogroups" | Changed line 239 |
| Line 278: Please be careful using incidence rather than prevalence. Incidence always includes a time period e.g. XX cases/ 100000 individuals/year. I don't think any of these animal studies describe incidence. | Changed line 294 |
| Lines313-314: Please include the references | Referenced |
| Supplementary Table 1 |  |
| This table needs to be checked. I am unclear what the serogroup (serovar) column indicates. I have checked a few references and for reference 2 (Allan et al 2020) the serogroups refer to those identified as causing human infections whereas reference 4 (Biggs et al 2011) appears to show all the serogroups included in the MAT. Only 8 of these serogroups were identified in human cases. I notice that in later references it is shown whether serogroups were not detected. Perhaps this was an oversight for earlier references? | The table has been modified to indicate which serogroups were examined in humans and animals and which were detected.  Related to your queries  Allan 2020 did not report the MAT results this was reported by Biggs 2011 and Maze 2018 so we have removed the MAT from this row  Biggs 2011 -all serogroups that were screened were detected (Table 4 of that paper). We have now highlighted the predominant groups detected to emphasise the important serogroups. |
| Under the PCR column some studies list DNA fingerprinting - this isn't PCR. | We have removed this |
| There is also reference to TAC. I'm not sure what this is? Perhaps it should be defined in the footnotes? | Defined in the footnotes |
| In addition, for some references e.g. 22 the results from hosts are shown in the PCR column. | For reference 22, there are both MAT and PCR results The PCR results 1/20 rodents positive and 2/7 of insectivores (Shrews) positive is recorded in the PCR column. |
